# Exploring the Genetic Background of the Differences in Nest-Building Behavior in European Rabbit

**DOI:** 10.3390/ani10091579

**Published:** 2020-09-04

**Authors:** Ildikó Benedek, Vilmos Altbӓcker, Attila Zsolnai, Tamás Molnár

**Affiliations:** 1 Institute of Environmental Sciences and Nature Conservation, Szent István University, Kaposvár Campus, 7400 Kaposvár, Hungary; altbac@gmail.com; 2 Research Institute for Animal Breeding, Nutrition and Meat Science, 2053 Herceghalom, Hungary; attila.zsolnai@gmail.com

**Keywords:** progesterone receptor, maternal behavior, hay carrying, heritability

## Abstract

**Simple Summary:**

The rabbit is one of the genetically most diverse farm animals, where domestication has resulted in a change primarily in the genes responsible for behavior. The elements of intensive production technology (e.g., nursing, change of nest material) can have a significant effect on maternal behavior. Its individual variability is evident in the timing of nest building and nest composition. As a result that the hormone progesterone strongly influences the steps of nest building, we investigated the association of genetic mutations in the progesterone receptor gene with nest-building behavior in wild-type rabbit does. In addition to the already described point mutations in domesticated lines, we detected a new mutation in our wild type rabbits. However, the timing of nest building (hay carrying) was related to an already described point mutation. The heritability of this trait was low, but it confirms the genetic determination of the behavior besides the environmental factors. One of the genotypes was responsible for the two days earlier initiation of the hay carrying behavior. The early initiation of this process allows animals to construct a high-quality nest resulting in better survival of the offsprings in nature.

**Abstract:**

Once a day, nursing and absentee mothering make the wild rabbit (*Oryctolagus cuniculus*) an ideal model animal for measuring differences in maternal behavior. Behavioral events and their hormonal regulation leading to parturition are well documented; however, the genetic background behind individual differences in this complex process is unknown. Decreased progesterone hormone level and the reduction of progesterone receptor activity are crucial to initiating the collection of nest material. The progesterone receptor gene is a likely candidate affecting nest-building behavior. In addition to several known point mutations in the progesterone receptor gene of the European wild rabbit, we have found a new mutation in the promoter region of the gene at 2682 T > C. Although this new single nucleotide polymorphism (SNP) was not involved in the formation of the nest-building behavior, an SNP (2464G > A) already described in the promoter region showed an association with individual differences in the initiation of hay carrying. The distribution of this SNP delivered an opposite result compared to domestic rabbits. Genotype (GG) with high uterine capacity was most frequent; the hereditary value of the trait was h^2^ = 0.10. Thus, progesterone receptor gene polymorphism may manifest in individual differences affecting breeding success in this species.

## 1. Introduction

Association analysis based on a candidate gene is widely used for detecting the genetic background of behavior. The connection between the variants in the mineral corticoid receptor gene and depression was already described in 2011 [1]. Oxytocin receptor gene (OXTR-gene) variations were linked to the regulation of mother–offspring binding in humans [2]. In the case of dogs, the oxytocin receptor gene variants could be linked to the social behavior towards humans [3]. In addition, the polymorphisms in the dopamine system seemed to clarify the differences in kinetic activities [4,5]. The variety of mating behavior of male and female Zebra finches (*Taeniopygia guttata*) could be related to the polymorphisms of the estrogen receptor gene (ESR) [6].

The maternal behavior of both the wild (*Oryctolagus cuniculus*) and the domesticated rabbit is limited to nest building for the offspring, and the daily nursing after parturition [7,8]. The behavioral steps follow a specific sequence and are regulated by both external [9,10,11], such as visual [12] and internal stimuli, such as hormonal factors [13,14,15]. The first step is nest-digging, which is triggered by the high β-oestradiol and progesterone levels on the 25th–26th day of gestation [16]. Due to the decreasing progesterone level, the digging ends, which triggers the carrying of nesting material 1–3 days before parturition [11,13,14]. The prolactin hormone level increases before parturition enabling the mothers to pull their fur, which is used for lining the nest [17].

Explaining the differences documented in the rabbit maternal behavior is a complex issue. Since nest-building gradually improves until the 3rd parturition [18], besides its supposed genetic background, it is heavily dependent on environment and experience. It has already been proven in domesticated rabbit lines that several aspects of reproduction, such as fetal survival, depends primarily on the maternal genotype [19]. In the case of the house mouse, the building of a sleeping nest has been proven to be a hereditary trait [20,21]. So far, the timing of nest-building has been considered a controversial trait in the rabbit, it is genetically encoded, and its expression can be modified by breeding practice [18]. Seltmann et al. [11], however, found nest-building flexible when consecutive reproduction periods were monitored.

The progesterone hormone has its impact via receptors (PR) located on the cell surface. The operation and efficiency of a receptor depend on which variant of the coding gene is carried by the given individual. By direct sequencing of the promoter region and the exon 1-8 of the progesterone receptor gene, six single-nucleotide polymorphisms (SNP) have been identified [22]. Four of these SNPs were segregated to 2 haplotypes. From this, the point mutation 2464G > located in the promoter region explained the difference between the lines with low and high uterus capacity. Peiro et al. [23] have already recorded the forming of two isoform products (PR-A and PR-B) in connection with the progesterone receptor gene in rabbits. They found that both isoforms are formed in the oviduct and the uterus and in mothers with high uterine capacity, but the expression of both products (PR-A and PR-B) is lower. In this line of mothers, the frequency of GG alleles was significantly higher at the position 2464G > A in the promoter region [23].

Progesterone receptors (PR) are located not only in the genitals but in the forebrain, where in the preoptic region, they act as a key in switching on and off the activation of nest building [24]. The function of PR has not been cleared yet, but Caba et al. [24] have recorded that the PR connected to progesterone metabolite stimulates the appearance of hay carrying behavior. It was also concluded that the reduction of progesterone receptor activity could be the triggering signal of carrying nest material [18].

The progesterone hormone may affect the reproduction success of the mother directly by modifying the embryonic development and indirectly through altering maternal behavior (nest building). Using wild-type rabbits kept under controlled conditions enabled us to quantify both the differences in maternal behavior and the changes in hormone levels and relate them to the genetic background. We wanted to know to what extent the polymorphism in the progesterone receptor gene (PGR gene) is associated with the timing of nest-building behavior.

## 2. Materials and Methods

### 2.1. Ethical Approval

The research was approved by the Committee on the Ethics of Animal Experiments of the Kaposvár University (permit number: MÁB/2-2/2019). The authors declare that all experiments were performed in accordance with the approved guidelines and regulations.

### 2.2. Test Animals

The tests were carried out on 30 mature breeding, 10–12-month-old European Rabbit does (Oryctolagus cuniculus), whose first farrowings were compared. In two cases, the hormone level determinations were incomplete, so all the other data coming from these animals were excluded from the experiment. The rabbits derived from captured wild rabbits, whose offsprings were kept in cages. The animals were mated by natural breeding. The tested animals had been imprinted during the first week of lactation after birth [25] to ensure secure handling [26] by reducing their fear of humans.

### 2.3. Housing

Animals were housed individually in cages supplied with farrowing boxes (40 × 25 × 31 cm). The size of a cage (80 × 60 × 45 cm) complies with the legislation in force. The cages were made of welded wire mesh, supplied with hand-refillable feeders and hay racks on the front wire. In addition, there was a galvanized steel sheet litter tray running on rails under each cage. Hand-refillable drinkers provided access to water. The rabbits had ad libitum access to water, hay and commercial rabbit fodder. The environmental conditions in the rabbit house were the following: the temperature: 15.4 ± 1.6 °C, relative humidity: 57.8 ± 8.5%, wind: 13.2 ± 3.5 km/h.

### 2.4. Studying Behavior

In order to study the grass collecting behavior during nest building, we provided dried grass in addition to the usual hay as nest material six days before the expected parturition (from day 25). We recorded every 12 h when the pregnant rabbit started carrying grass. We ranked the nests according to the weight of fur and hay, and also according to the rate of fur and hay. When the nestlings had reached the age of 21 days, the nest was removed from the farrowing box. The nest was wet from urine, so we dried it first. After weighing the complete nest, we mixed the fur and grass, evenly to get a homogenous mixture, and took ten samples (appr. 1–1.5 g each). All samples were sorted into fur and hay fibers, and each group was weighed on a Sartorius scale to two decimal places weight to the nearest gram. The rate of hay, and fur weight, helped to estimate the amount of the fur and hay in the complete nest.

### 2.5. Determining Hormones from Feces

We carried out measurements of the progesterone and cortisol levels from feces on the basis of metabolites [27,28]. We provided a completely clean tray for the rabbits before collecting, and then the droppings were collected after 24 h after parturition. Fecal glucocorticoid metabolites (GCMs) were extracted from the droppings, and the samples were stored at −20 °C. Following a feces extraction protocol [29], we put 200 mg feces into glass vials. The hormone metabolites were extracted with 1.6 mL 80% methanol and 200 µL distilled water. The vials were capped and were vortexed for 30 min. The samples were centrifuged (2450 rpm, 20 min, 4 °C); the supernatant liquid was removed and stored at −55 °C. At the time of use, the samples were dried in a chamber (Binder), and then they were solubilized with inhibin assay A, B buffer (ASB) in dilution 1:1. During processing cortisol and progesterone measurements, we used a direct radioimmunoassay (RIA) test. This method has been developed to determine animal blood plasma hormone in rabbits by using tritium labeled hormones (cortisol and progesterone-1,2,6,7-3H (N)) and polyclonal antibodies with high specificity cortisol-21-HS-BSA and 11aOH progesterone 11HS. The sensitivity of the test was 433.4 ng/g in the case of cortisol, and it was 199.26 ng/g in the case of progesterone. The value of the intra-assay coefficient was ˂ 5 CV% in the case of both cortisol and progesterone, while the inter-assay coefficients of were 9.63, and 1.68 CV% in the case of progesterone and cortisol, respectively.

### 2.6. Sequencing PGR

Extraction of the DNA was carried out from fur samples (MÁB/2-2/2019), by cutting the hair roots and with the help of 5% Chelex resin [30]. The isolation was carried out according to the standard procedure. We gained 400 µL purified DNA solution; concentration was set to 55 ng/µL. A 558 bp long segment from the promoter region of the PGR gene in genomic DNA was amplified by the primers and conditions described by Peiro et al. [22]. These conditions were as follows: 10 min at 95 °C, followed by 35 cycles, 30 min at 95 °C, 60 s at 66 °C, 90 s at 72 °C. The final extension step was 15 min at 72 °C. The primers had universal M-13 tails, which enabled the annealing of sequencing primers. The final volume of the reaction mixture was 25 µL, which contained the following components: 1.2 µL genomic DNA solution (55 ng/µL), 12.5 µL 2× Platinum Superfi Master Mix, 5 µL 5× Enhancer, 1.25 µL PGR-F and PGR-R primers (10 µM in stock solution). The resulting 558 bp long product went through silica membrane purification and was used for sequencing reaction with the help of a BigDye terminator 3.1 sequencing kit (ThermoFisher Scientific). The temperature profile of the sequencing reaction was as follows: 96 °C for 3 min, 96 °C for 10 s, 55 °C for 20 s, 60 °C for 1 min, 15 s, then 4 °C. The final volume of the reaction mixture was 10 µL; its components were: 0.8–2 µL sample, 1.4 µL BigDye reaction mix, M-13 sequencing primer, distilled water as described in the manual. Sequencing was carried out with the help of an ABI 3100 genetic analyzer (Applied Biosystems). The sequences of the progesterone receptor gene of the 30 mother rabbits were aligned to the gene bank sequence (identification number X06623.1) with the program Clustal Omega [31].

### 2.7. Statistical Processing

Genetic diversity was determined by calculating the observed heterozygosity (Ho) and unbiased expected heterozygosity (He), the effective number of alleles (Ne) and by testing the Hardy Weinberg equilibrium in each SNPs using GENALEX version 6.5 [32,33]. Polymorphism information content (PIC) was calculated by CERVUS 3.0.7 software [34]. Linkage disequilibrium data were analyzed with the DNAsp 5.10 program [35]. To test whether there was an association of hay carrying behavior and hay weight with the PGR polymorphisms, we used a general linear model (GLM) procedure implemented in SPSS 17.0 (SPSS Inc., Chicago, IL, USA, 2008), according to the following model: dependent variables were, time of hay carrying and hay weight, fixed factors were SNP 2464, SNP 2682 and SNP 2866 genotypes, covariants were the progesterone and cortisol hormone levels at the day of parturition. Partial eta squared was calculated to determine the effect sizes of the factors. Two-step cluster analysis was used to assign the rabbit does into groups according to the start of stay carrying, and chi-squared test (linear by linear association test) was used to determine the significance of the difference in the genotype distributions of groups. Narrow sense heritability of the SNP 2464G > A was calculated by the standard procedure cited by Falconer and Mackay [36] with the following equations h^2^ = V_A_/V_P_ where V_A_, the additive variance and V_P_ is the phenotypic variance. V_A_ was calculated by the equation V_A_ = 2pqα^2^ where α = a + d (q − p) and p and q are the frequencies of the two alleles, a is the genotypic value of the homozygote and d is the genotypic value of the heterozygote.

## 3. Results

### 3.1. Identification of Point Mutations

**Figure 1:**
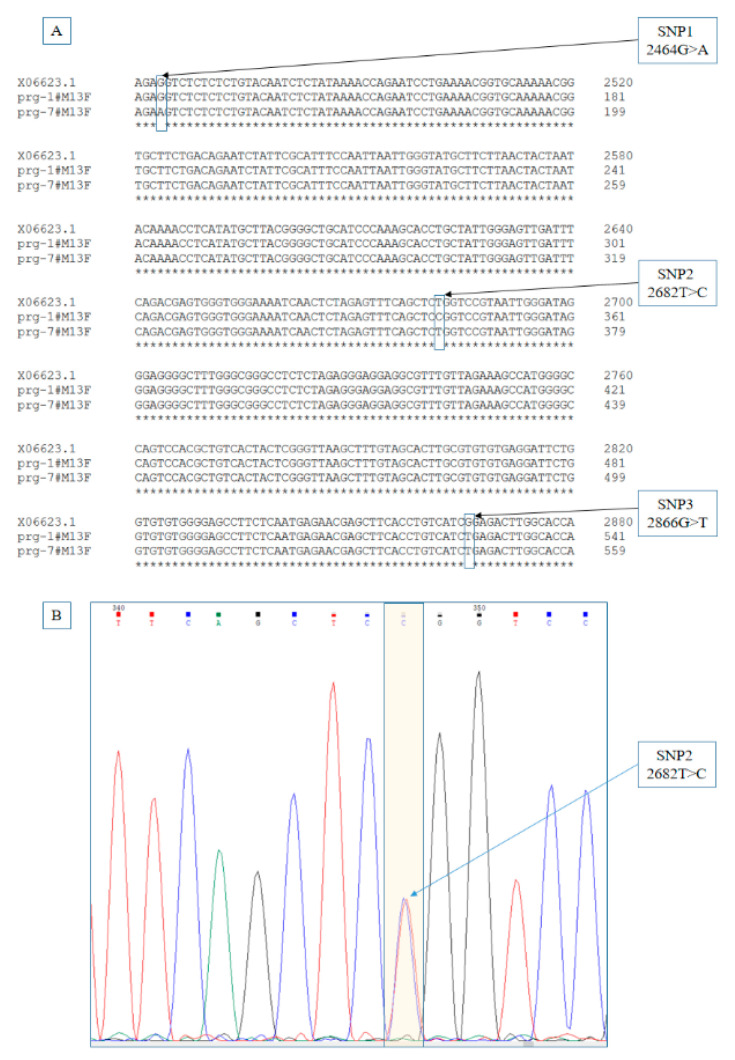
Location of the new single-nucleotide polymorphism (SNP) (2682T > C) in the promoter region of Progesterone Receptor Gene (PRG) sequence.

Based on the sequencing, the original point mutation has been detected at location 2464G > A in the promoter segment and 2866G > T in exon 1 [22]. At 2682T > C in the promoter region, a new point mutation has been identified (Figure 1).

X06623.1 is the reference sequence from the GenBank. The mutations are highlighted with a frame. B part represents the part of the chromatogram of the sequence containing the SNP 2 in a heterozygote individual.

### 3.2. Examine of Genotype Distributions

Table 1 shows the distribution of observed genotypes, observed heterozygosity (Ho), expected heterozygosity (uHe), effective allele size (Ne) and PIC value. Examining the distribution of genotypes indicates that they are identical with the Hardy–Weinberg equilibrium in the case of all three SNP (P > 0.05, Table 1). The PIC values indicate that the rabbit stock shows mediate polymorphism.

### 3.3. The Linkage Between Point Mutations

Table 2 shows the relationships of SNPs in the promoter region and on exon 1. Neither of the SNP pairs showed significant linkage disequilibrium (LD). Based on our results, there is no significant linkage disequilibrium between any of the pairs made up 2 SNPs (2464G > A and 2682T > C) in the promoter region and one on exon 1 (inherited independently).

### 3.4. The Relationship between Hay-Carrying Behavior, the Amount of Hay in the Nest and Polymorphisms in the PGR Gene

The raw data of the parameters measured in the study and the descriptive statistics of the timing of nest construction and the composition of the nest can be found in Table S1 and Table 3.

The starting time of hay carrying was significantly affected by several factors, such as the two hormones (progesterone and cortisol) and 2464G > A SNP located in the PGR gene (Table 4). Neither the impact of the other two SNPs nor the interactions have been proven significant. We detected a considerable impact concerning the hay weight at interactions of SNP2-3; however, the impact of the individual SNPs was not significant.

### 3.5. Allele Distribution in Groups Formed as a Result of Behavioral Cluster Analysis and Heritability

**Figure 2:**
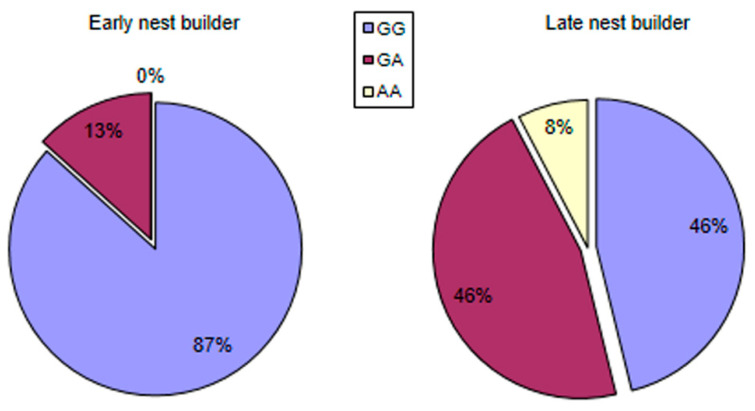
Distribution of the PRG 2464G > A SNP genotypes in the groups clustered by the time when hay collecting started.

In the cluster analysis, the rabbit does were classified into two clusters according to the time when hay carrying started. Fifteen individuals were classified into the Early group (time of hay carried 3.6 ± 0.7 days before parturition). In comparison, thirteen individuals were classified into the Late group (time of hay carried 0.8 ± 0.5 days before parturition). The Early group included GG genotype to 87%, heterozygote alleles (GA) to 13%; (Figure 2), but it did not contain any genotype AA individuals. In the Late group, the GG ratio decreased significantly, the GA ratio increased, significantly, and also genotype AA was present in this group (Linear by linear association χ^2^ = 5.184, df = 1, P = 0.023). In genotype GG individuals, the starting time of hay carrying was 2.7 ± 0.3 days; in the case of genotype GA, it was 1.5 ± 0.4 days, and it was 0.5 day in genotype AA does. In the case of point mutation 2464G > A the calculated additive genetic variance was (V_A_ = 2pq*α^2^, α = 0.909) V_A_ = 0.2444. Based on h^2^ = V_A_/V_P_ (Vp = 2.353), the heritability of point mutation PGR 2464G > A is h^2^_PGR2464_ = 0.10.

## 4. Discussion

We studied the association between maternal behavior, hormonal levels and genetic background in wild-type rabbits. Among the studied variables, the effect of progesterone and cortisol proved to be significant. Decreased progesterone levels have long been known to trigger nest-building behavior [17]. Changes in fecal cortisol levels are a good indicator of environmental stress in rabbits [27]. In wild populations, a delay in nest-building behavior has been described for subordinate does, which was explained by the effect of social stress [11]. As a result that does were placed individually in our study, and interactions between individuals were thus limited. During the pregnancy and the parturition there were no extreme changes in the environmental conditions and the does were imprinted to humans to reduce fear. The captive environment could have long term impact on the individuals, which could manifest in elevated cortisol levels [37,38]. The experiment was carried out on F2 captive-born, wild rabbit genotype resulting in that does were not selected for the captive environment and showed high behavioral variability. As a result that cortisol plays a role in the regulation of parturition in many farm animal species [39], it can be hypothesized that rabbit nest-building behavior is also affected by individual stress sensitivity. Exploring the impact of environmental stress on behavior requires further studies. We found a new point mutation in the promoter region at 2682T > C located only 218 base pairs from the already known SNP (2464G > A) described by Peiro et al. [22]. 2682T > C did not have any impact on the elements of nest-building observed in our experiment; however, it indicates how diverse is the genetics of the European wild rabbit compared to domesticated lines.

The distribution of polymorphisms in the progesterone receptor gene can help to understand the differences in rabbit reproduction. In the case of point mutation 2464G > A in PGR gene, genotype GG was 67.85%, genotype GA was 28.57%, while genotype AA was 3.57% in our tested stock (see Table 1). Contrary to our results, genotype heterozygote (GA) was dominant in domesticated breeds [22,40,41]. However, the frequency of genotype GG and AA differed between the lines. In the stock selected for uterine capacity [22], the advantage of GG genotype was described (32.9% GG, 17.1% AA) in contrast to Egyptian and French rabbit lines, where genotype AA was most frequent. These lines also involved indigenous breeds and lines selected for body weight [40], and New-Zealand white rabbit lines [41] had similar results. This indicates that the genotype distribution can differ in domesticated lines and the wild-type rabbits in our study. This could result from the fact that in domesticated lines, selection for body weight could have affected the PGR gene negatively. The main limiting factor in the evolution of litter size is the prenatal loss, which can account for up to 30% [42]. As the majority of wild rabbit mothers are capable of having three breeding seasons on average under natural conditions [10], the number of remaining nestlings is vital for the fitness of the mother. Therefore, the genotype with high uterine capacity (GG) will be adaptive.

In the case of our wild-type stock, the value of observed heterozygosity (Ho = 0.286) was lower than that of the expected heterozygosity (uHe = 2.299). However, it did not show any significant difference from the Hardy–Weinberg equilibrium. These values were reversed in rabbit stocks examined by El-Aksher et al. [40]. In all four cases, the Ho values were higher, and the populations differed significantly from the Hardy–Weinberg equilibrium (Table 1). The polymorphism information content (PIC = 0.25) can be considered to be moderate, similarly to the stocks mentioned above (PIC = 0.367) in the study carried out by El-Aksher et al. [40].

We did not find significant linkage disequilibrium between the two point mutations (2464G > A and 2682T > C) in the promoter region and the point mutation in exon. However, Peiro et al. [22] was able to separate two haplotypes based on SNPs in 2464G > A and exon 1 in rabbit stocks selected for low and high uterine capacity. Our results indicate that the polymorphism (2464G > A) in the progesterone receptor gene affects the nest-building behavior regarding the timing of hay carrying. The Early group starts carrying hay in the nest nearly three days (2.8) earlier, and typically most of them have genotype GG (87%). While in the Late group, where the mothers start hay carrying only a few hours before parturition, the frequency of A allele is increased. Concerning the amount of collected hay, there was no significant difference between the Early and Late groups. This implies that the nest quality (concerning the amount of hay) of early nest builders is similar to that of late nest builders if the animals are offered ad libitum hay. This condition is quite the opposite to natural situations where hay is scattered, and collection is time-consuming [9]. The survival of the pups in the first few days is determined by the nest structure (insulating ability) [43] and the thermoregulatory capacity of the pups, as well as the ambient temperature [44]. Due to less practice and stronger social stress, does with lower social rank can build a less suitable nest [45], which is also reflected in higher pup mortality under natural conditions.

As shown in Figure 2, mothers genotyped on the basis of SNP1 differ in the starting time of hay carrying. Mothers with GG alleles started hay carrying 2.8 ± 0.3 days before parturition, mothers with genotype GA started it 1.5 ± 0.3 days earlier, while does with genotype AA started to carry hay only half a day before parturition. This difference is clearly visible when looking at the distribution of genotypes in mothers sorted by the starting time of hay carrying (Early and Late group) as well. In the Early group, GG allele was 87%, while GA allele was only 13%. In the late group, however, GG allele was only 46%, and the rate of heterozygotes made up a significantly higher value, 46% (see Figure 2). There are abundant examples in both human and animal life that the polymorphisms in receptor genes modify behavior [1,3,4,5,6,46,47]. This receptor (PR) has a significant impact on the behavior of mothers since it is connected to the starting and finishing of the nest-building behavior [24]. The point mutation examined in our experiment influences other maternal traits as well. It explains the differences between the lines with low and high uterine capacity on the basis of the genotype frequency [22], even at the gene expression level [23].

We assessed the heritability of point mutation PGR 2464G > A as h^2^ = 0.10. Concerning the maternal behavior of rabbits, the heritability of several variables of reproductive biology parameters has been examined so far. Among these, the litter size and the length of gestation can be connected to the progesterone receptor. The length of gestation is a weakly hereditary trait with an h^2^ value between 0.00 and 0.06 [48,49]. Concerning litter size, the h^2^ value of live-born nestlings accounts for 0.12–0.14 of variance in the first gestation [50,51]. These values are similar to our results. The hereditary value of the laboratory mouse (Mus) nest-building behavior was 0.14–0.21 [52], which is slightly higher than our value, but this refers to a different species and to building a sleeping nest. The h^2^ value of birds (blue tit (*Cyanistes caeruleus*)) concerning nest size was 0.12 [53], which equals approximately with our results.

The specialized maternal behavior and the available laboratory stock of wild-type rabbits enabled us to focus on crucial parts of maternal behavior. We could determine the genetic background contributing to the variation in nest building. The idea that differences in nest building can have genetic causes is suggested by Walsh et al. [54] for weaver birds. The relation described by us complies with the differences found in the nest-building activity of diverse mouse lines [20]. However, study conditions with less restricted environmental variation may also be needed for a full understanding of the factors affecting reproduction.

## 5. Conclusions

The primary aim of the present study was to evaluate the role of the candidate PGR gene polymorphisms in the nest-building behavior of rabbits. We demonstrated that SNP (2464G > A) has a heritable effect on the timing of the behavior. Our study highlights that differences in nest quality, unlike those observed under natural conditions, do not occur with an unlimited amount of available grass. This raises the possibility of the accumulation of maladaptive genotypes during rabbit breeding, depending on the technology used. In addition, it would be worthwhile to study the adaptability of the trait under different ecological conditions in order to understand the evolution of the behavior better.

## Figures and Tables

**Table 1:** Genotypic distribution and genetic diversity in 3 SNPs located on the PGR.

SNP	Observed Genotype						Ho	uHe	HWE		Ne	PIC
									χ^2^	Prob.		
2464G > A	GG	19	GA	8	AA	1	0.286	0.299	0.019	0.890	1.415	0.250
2682T > C	TT	17	TC	11	CC	0	0.393	0.321	1.673	0.196	1.461	0.266
2866G > T	GG	13	GT	14	TT	1	0.500	0.416	1.418	0.234	1.690	0.325

Ho: observed heterozygosity, uHe: unbiased expected heterozygosity, Ne: effective allele size and PIC: Polymorphism information content, HWE: the Hardy–Weinberg equilibrium, Prob: probability.

**Table 2:** Allele and haplotype frequency distribution and linkage disequilibrium in the case of tested SNPs.

	Allele Frequency		Haplotype Frequency		D’	r	χ^2^	P
**SNP1-2**	G	0.82	GT	0.67	0.018	0.016	0.029	NS
	A	0.18	GC	0.16				
	T	0.80	AT	0.13				
	C	0.20	AC	0.03				
**SNP1-3**	T	0.80	TT	0.56	−0.632	−0.181	3.651	NS
	C	0.20	TG	0.24				
	T	0.71	CT	0.15				
	G	0.29	CG	0.04				
**SNP2-3**	G	0.82	GT	0.55	−0.239	−0.077	0.662	NS
	A	0.18	GG	0.27				
	T	0.71	AT	0.16				
	G	0.29	AG	0.02				

SNP1-2464G > A, SNP2-2682 T> C, SNP3-2866G > T.

**Table 3:** Descriptive statistics of the traits measured in the experiment.

Trait	N	Mean	S.D.	Min	Max
**Time of Hay Carrying (Day)**	28	2.3	1.5	0.5	5.0
**Hay Weight (g)**	28	169.2	61.5	90.5	331.1
**Rate of Fur (%)**	28	18.9	16.4	3.7	74.5

**Table 4:** Hay carrying behavior and hay weight in relation to polymorphism of PGR 2464G > A, 2682T > C, Exon 1.

	df	Hay Carrying Behavior				Hay Weight			
		Mean Square	F	P	Partial Eta Squared	Mean Square	F	P	Partial Eta Squared
**Intercept**	1	**10.94**	**6.877**	**0.018**	**0.288**	**85041.200**	**21.614**	**0.001**	**0.560**
**Progesterone**	1	**9.873**	**6.203**	**0.023**	**0.267**	1473.090	0.374	0.549	0.022
**Cortisol**	1	**8.298**	**5.214**	**0.036**	**0.235**	2543.257	0.646	0.433	0.037
**SNP1**	2	**7.085**	**4.452**	**0.028**	**0.344**	1132.699	0.288	0.753	0.033
**SNP2**	1	0.299	0.188	0.670	0.011	1914.580	0.487	0.495	0.028
**SNP3**	2	1.595	1.002	0.388	0.105	236.410	0.060	0.942	0.007
SNP1 * SNP2	1	0.129	0.081	0.780	0.005	5301.626	1.347	0.262	0.073
SNP1 * SNP3	1	0.035	0.022	0.883	0.001	5662.326	1.439	0.247	0.078
SNP2 * SNP3	1	0.333	0.209	0.653	0.012	**18,082.703**	**4.596**	**0.047**	**0.213**

Generalized linear model: covariates: cortisol and progesterone, fix factors: SNP1-2464G > A, SNP2-2682T > C, SNP3-2866G > T. Significant factors are highlighted in bold.

## Supplementary Materials

The following are available online at https://www.mdpi.com/2076-2615/10/9/1579/s1, Table S1: The raw data of the experiment.
