# Supplementary material for: Exploring the Genetic Background of the Differences in Nest-Building Behavior in European Rabbit
Source: Animals (Basel). 2020 Sep 4;10(9):1579. doi: 10.3390/ani10091579 (PMC7552269; doi:10.3390/ani10091579)
Supplement: Supplementary file 1 [file animals-10-01579-s001.zip › supplemengtary material/Table S1.pdf]

Table S1
The raw data of the experiment.
Title:Exploring the genetic background of the differences in nest-building behavior in European rabbit
Authors: Ildikó Benedek*, Vilmos Altbäcker, Attila Zsolnai, Tamás Molnár*

does	Time	Hay weigh	progesterone	cortisol	2464G>A	2682T>C	2866G>T	cluster
1	3	133,82	2215,44	1952,99	GA	TT	TT	2
2	3	205,38	131,45	454,54	GG	TC	TG	2
3	2	91,44	738,69	781,85	GG	TT	TG	1
4	0,5	87,21	703,78	1744,93	GG	TT	TG	1
5	3	155,8	311,01	459,97	GG	TT	TG	2
6	0,5	135,55	656,3	1099,37	GG	TT	TG	1
7	3	209,69	472,65	1488,66	GG	TT	TT	2
8	4	102,78	1215,74	1004,77	GG	TC	TT	2
9	3	132,07	130,82	406,69	GG	TC	TT	2
10	3,5	160,57	254,72	372,98	GG	TC	TG	2
11	0,5	184,63	354,09	630,7	GA	TC	TT	1
12	0,5	151,33	283,34	1100,82	GG	TT	GG	1
13	0,5	171,94	817,31	2124,8	GA	TT	TT	1
14	3	134,48	795,29	564,73	GG	TT	TG	2
15	1,5	267,13	425,48	736,9	GA	TT	TG	1
16	0,5	106,73	203,15	371,53	GA	TT	TG	1
17	3,5	98,58	560,7	695,58	GG	TT	TG	2
18	5	234,28	923,28	642,8	GG	TT	TG	2
19	5	325,4	861,65	1041,01	GG	TT	TT	2
20	4,5	298,73	776,74	462,91	GG	TT	TT	2
21	1,5	84,43	481,14	474,04	GA	TC	TT	1
22	0,5	206,68	590,57	637,91	AA	TT	TT	1
23	0,5	133,93	170,44	723,13	GG	TT	TT	1
24	3	165	265,41	498,54	GA	TC	TT	2
25	1	129,39	898,13	734	GG	TC	TG	1
26	3	82,02	461,01	668,39	GG	TC	TG	2
27	4,5	159,06	977,06	716,24	GG	TC	TG	2
28	1,5	214,15	465,73	507,46	GA	TC	TT	1

Time: Start of the hay carrying behaviour (days before parturition), Hay weight (g)
progesterone: faecal progesterone metabolite content (ng/mg),
cortisol: faecal cortisol metabolite content (ng/mg)
2464G>A: SNP in the promoter region (Peiro et al 2008), 2682T>C: SNP in the promoter region (new)
2866G>T: SNP in Exon 1 (Peiro et al 2008),
cluster: clusters according to the time when hay carrying started:(1 Late gorup 2 Early group)
